# Enhanced etch characteristics of EUV PR masked SiON through the ion beam grid pulsing technique

**DOI:** 10.1038/s41598-025-04632-x

**Published:** 2025-06-06

**Authors:** Hae In Kwon, Yun Jong Jang, Kyoung Chan Kim, Hong Seong Gil, Ju Young Kim, Seong Hyun Ryu, Do Seong Pyun, Dae Whan Kim, Woo Chang Park, Ji Yeon Lee, Jin Woo Park, Sang Wuk Park, Geun Young Yeom

**Affiliations:** 1 School of Advanced Materials Science and Engineering, Sungkyunkwan University, Suwon, 16419 Republic of Korea; 2 SKKU Advanced Institute of Nano Technology (SAINT), Sungkyunkwan University, Suwon, 16419 Republic of Korea; 3 Department of Photovoltaic System Engineering, Sungkyunkwan University, Suwon, 16419 Republic of Korea; 4 Department of Semiconductor Display Engineering, Sungkyunkwan University, Suwon, 16419 Republic of Korea; 5 School of Chemical Engineering, Sungkyunkwan University, Suwon, 16419 Republic of Korea; 6 Advanced Process Development, Semiconductor R&D Center, Samsung Electronics Co. Ltd., Hwaseong, 18448 Republic of Korea

**Keywords:** Ion beam etching, EUV PR, SiON, Grid pulsing, LER, Etch selectivity, Engineering, Materials science, Nanoscience and technology

## Abstract

EUV lithography technology, applied in nano-patterning processes, enables the creation of fine patterns below 10 nm. However, issues still remain due to the reduced etch selectivity and increased line edge roughness (LER) caused by the thin thickness and weakness of organic EUV photoresist (PR). In this study, research was conducted to improve the low etch selectivity and high LER using a novel grid pulsed ion beam etching technique. In this system, Ar/H_2_ plasma is generated in the inductively coupled plasma (ICP) source, and the Ar^+^/H_x_^+^ ion beam is irradiated while fluorocarbon gas is injected into the process chamber. Grid pulsing technique increases the etch selectivity of SiON over EUV PR while improving LER. With a 50% duty ratio and optimal gas flow conditions (Ar:H_2_ ratio = 1:3 to the ion beam source and CF_4_:C_4_F_8_ = 1:1), the etch selectivity of SiON over EUV PR approached ∞ while maintaining the LER close to the reference.

**Supplementary Information:**

The online version contains supplementary material available at 10.1038/s41598-025-04632-x.

## Introduction

As the semiconductor industry faces critical challenges in patterning at ≤ 10 nm scales, photolithography is transitioning from ArF immersion technology to extreme ultraviolet (EUV) technology^1–5^. This shift occurs because EUV technology, utilizing a 13.5 nm wavelength light source instead of a 193 nm wavelength source, provides cost advantages with fewer process steps compared to ArF immersion technology, which requires higher costs and complex process control due to multi-patterning^6–10^.

However, several challenges persist in EUV technology-based fine patterning. Due to the low sensitivity of the resist to EUV light, EUV resist typically has an extremely thin thickness of ≤ 50 nm. As the critical dimension of patterns decreases, a trade-off exists between resolution and EUV photoresist (PR) thickness, necessitating thinner resist layers. This thinning also decreases the risk of pattern collapse as it leads to lower aspect ratios^11^.

Consequently, challenges in etching processes to increase etch selectivity are intensifying due to reduced etch resistance^12,13^. Another significant challenge is line edge roughness (LER), which refers to the variation in edge positions of line/space patterns from ideally designed positions on the mask. Compared to ArF light sources, EUV photons possess higher energy, leading to approximately 14 × fewer photons being absorbed in the photoresist compared to DUV. This reduced photon count participating in edge definition increases LER due to stochastics (shot noise)^14–20^. The increased LER in EUV PR transfers to the final target material edge lines, causing pattern interruptions, misalignment among patterns in subsequent processes, and eventually degradation of device electrical properties ^13,21,22^.

Recently, to address problems related to EUV PR, research has investigated not only EUV lithography itself but also etching technology using EUV PR patterns. To improve etch selectivity and prevent PR wiggling during etching (thereby improving LER), a CF_x_ passivation layer was formed on EUV PR pattern sidewalls during etching with dual-frequency capacitively coupled plasma by employing the DC superposition effect^23,24^. To increase EUV PR etch resistance, a cyclic etching method has been investigated by alternating polymer deposition and dielectric etching during the process cycle^25^. As an EUV PR pretreatment method, EUV PR was treated with CS_2_ plasma before etching to improve etch resistance to fluorine-based etch chemistry^26^. Additionally, to improve etch resistance of EUV PR, neutral beam etching has been utilized instead of conventional reactive ion etching to avoid releasing ionization potential energy through ion bombardment on the EUV PR surface during etching^27^.

**Fig. 1:**
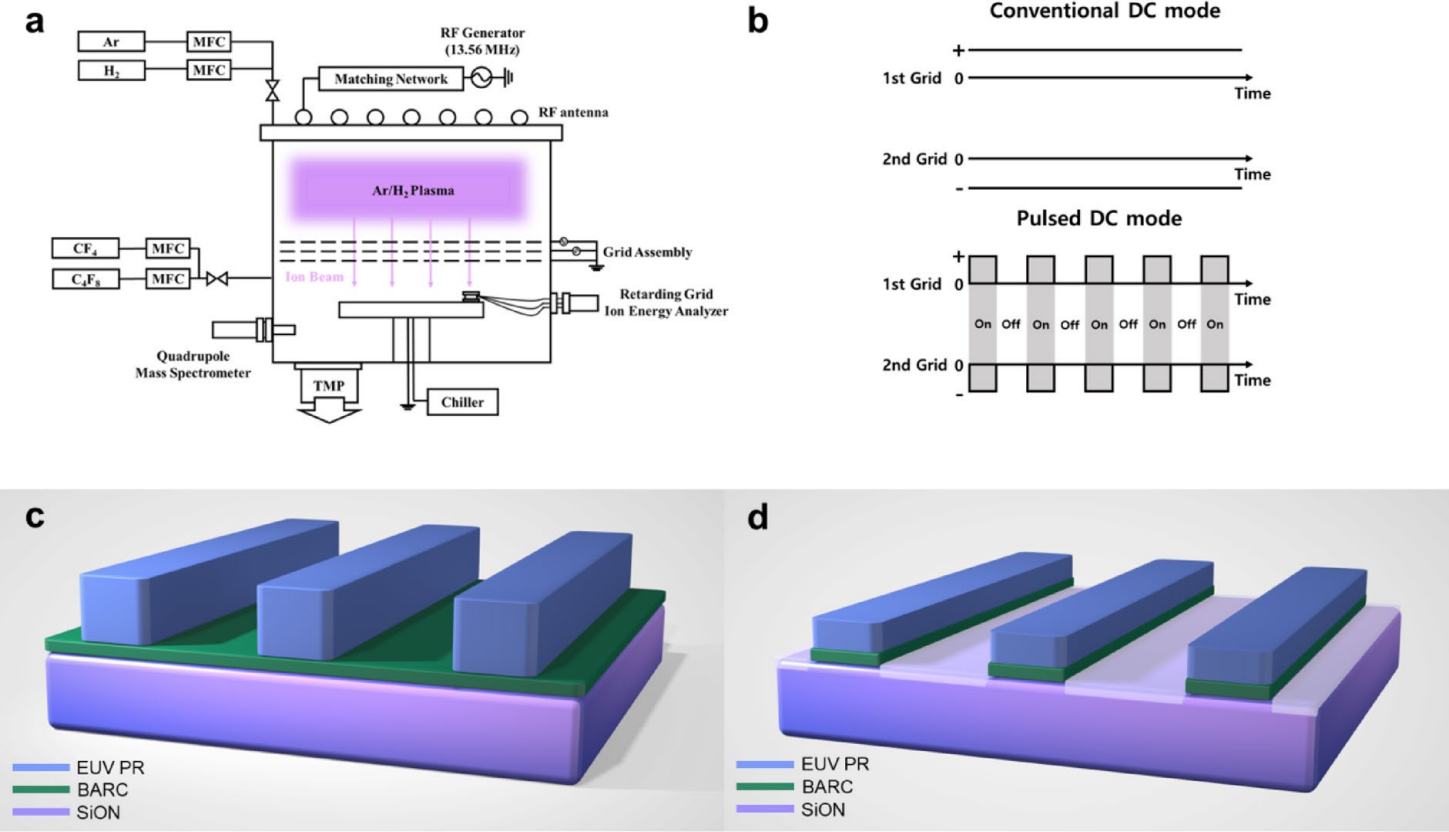
Grid pulsed system and the EUV PR pattern. (**a**) Schematic diagram of the ICP-type ion beam etcher with a 3-graphite grid assembly used for grid pulsed ion beam etching. The third grid was grounded. (**b**) The concept of grid pulsing mode during ion beam etching. (**c**) Schematic diagram of the EUV PR pattern structure before etching. (**d**) Schematic diagram of the EUV PR pattern after etching.

In this study, we investigated grid pulsed ion beam etching as a novel method for etching EUV PR-masked SiON to improve the etch selectivity of SiON over EUV PR and to decrease EUV PR LER. Figure 1a depicts the experimental setup, while Fig. 1b shows the system components for pulse application. Figure 1c,d provide information on the pattern used in the experiment, presenting the pattern before and after etching. To etch SiON selectively to EUV PR, a grid pulsed ion beam generated by an Ar/H_2_ plasma from a three-grid ion beam source was irradiated onto an EUV PR-masked SiON wafer while injecting C_x_F_y_ gas mixture into the process chamber located below the ion beam source. By controlling Ar/H_2_ ratios, pulse duty percentage of the ion beam source, and C_x_F_y_ gas mixture ratios in the process chamber, we investigated the effects of process conditions on SiON-to-EUV PR etch selectivity and LER variation.

## Results and discussion

### Ion beam etching with the continuous DC grid mode

**Fig. 2:**
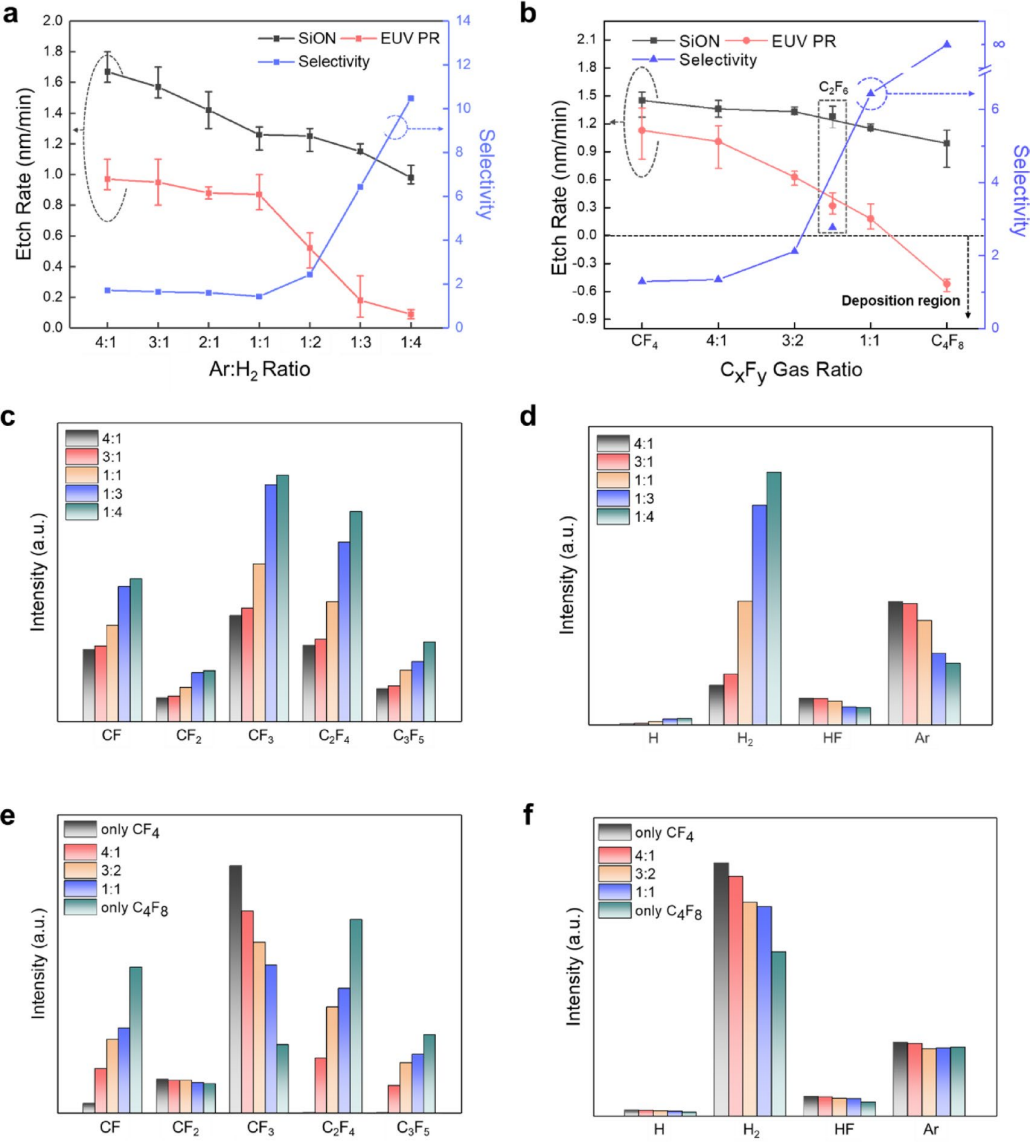
Etch characteristics and gas composition analysis for continuous DC grid mode. (**a**) Etch rates of EUV PR and SiON and their selectivity ratios as a function of Ar:H_2_ ratio (with CF_4_:C_4_F_8_ = 1:1). (**b**) Etch rates and selectivity as a function of the CF_4_:C_4_F_8_ ratio (with Ar:H_2_ = 1:3). The C_2_F_6_ is positioned separately between pure CF_4_ and the first CF_4_:C_4_F_8_ mixture ratio. (**c**) QMS analysis showing relative intensities of C_x_F_y_ species (CF, CF_2_, CF_3_, C_2_F_4_, C_3_F_5_) for different Ar:H_2_ ratios (4:1, 3:1, 1:1, 1:3, 1:4). d, QMS analysis of hydrogen-based gas (H, H_2_, HF) and Ar intensities for the same Ar:H_2_ ratios. e, Relative intensities of C_x_F_y_ species for different CF_4_:C_4_F_8_ compositions (only CF_4_, 4:1, 3:2, 1:1, only C_4_F_8_). f, Hydrogen-based gas and Ar intensities for the same CF_4_:C_4_F_8_ compositions.

Figure 2a,b shows the etch rates of SiON and EUV PR and the resulting etch selectivities measured as a function of (a) Ar:H_2_ ratio fed to the ion beam source and (b) CF_4_:C_4_F_8_ gas ratio fed to the process chamber below the ion beam source. A 13.56 MHz 300 W RF power was applied to the ICP coil of the ion beam source while operating the grid voltages continuously (+ 150 V to 1st grid, − 200 V to 2nd grid, and 0 V to 3rd grid) without pulsing. The ratio of the Ar:H_2_ gas mixture was varied from 4:1 to 1:4 at 2 mTorr, and the ratio of the CF_4_:C_4_F_8_ gas mixture was varied from 1:0 to 0:1 at 0.5 mTorr while maintaining the process chamber pressure at a total of 2.5 mTorr.

For Fig. 2a, the ratio of CF_4_:C_4_F_8_ was kept at 1:1. As shown, increasing the H_2_ percentage in the Ar:H_2_ gas mixture decreased the etch rates of both SiON and EUV PR; however, the etch selectivity of SiON over EUV PR increased from 1.7 (at Ar:H_2_ = 4:1) to 10.5 (at Ar:H_2_ = 1:4). Therefore, increasing the H_2_ percentage in the Ar:H_2_ gas mixture fed to the ion beam source consistently decreased the etch rate but enhanced the etch selectivity of SiON over EUV PR.

To further improve the etch selectivity of SiON over EUV PR, the ratio of C_4_F_8_ in the C_4_F_8_:CF_4_ gas mixture fed to the process chamber was varied while keeping the ratio of Ar:H_2_ fed to the ion beam source at 1:3 to maintain the etch rate of SiON higher than ~ 1.15 nm/min. As shown in Fig. 2b, with fixed Ar:H_2_ at 1:3, increasing the percentage of C_4_F_8_ in the CF_4_:C_4_F_8_ gas mixture (while maintaining 0.5 mTorr total pressure) slightly decreased the etch rates of both SiON and EUV PR. Simultaneously, the etch selectivity of SiON over EUV PR increased significantly from 1.3 (for CF_4_:C_4_F_8_ = 1:0) to near ∞ (for CF_4_:C_4_F_8_ = 0:1). When C_2_F_6_ was used instead of the CF_4_:C_4_F_8_ gas mixture (shown as a separate data point with a different marker in Fig. 2b), the etch rate and selectivity values were located between those of pure CF_4_ and pure C_4_F_8_. This indicates the importance of the C:F ratio in determining etch rates and selectivities, although the relationship is not strictly exactly proportional to the C:F ratio of the fluorocarbon gas.

The LER increased with increasing C_4_F_8_ ratios, producing rougher sidewall lines. When measured using the LACERM software, the LER increased from ~ 5.8 nm (for reference) to ~ 9.6 nm (for pure C_4_F_8_) (Supplementary Fig. S1). Therefore, despite achieving high or even infinite etch selectivity with higher percentages of C_4_F_8_, very high C_4_F_8_ ratios appear unsuitable for selective EUV PR-masked SiON etching due to LER degradation.

### Etch mechanism analysis in the continuous DC grid mode

To understand the variations in etch rates, selectivity, and LER, the dissociated gas composition in the process chamber was analyzed using Quadrupole Mass Spectrometry (QMS). The results are shown in Fig. 2c,d for the conditions in Fig. 2a and in Fig. 2e,f for the conditions in Fig. 2b (QMS raw data available in Supplementary Figs. S2 and S3, respectively).

Figure 2c,d shows the increasing H_2_ in the Ar:H_2_ gas mixture elevated H and H_2_ concentrations while decreasing Ar due to the changed gas mixture ratio. Additionally, we observed increases in CF_x_ (x = 1–3) and C_x_F_y_ species such as C_2_F_4_ and C_3_F_5_ dissociated from CF_4_ and C_4_F_8_, along with the formation of HF. These changes indicate more polymeric gas conditions for etching. While CF_3_ is a low C/F radical associated with etching, CF_x_(x = 1,2) and C_x_F_y_ species like C_2_F_4_ and C_3_F_5_ are high C/F ratio radicals related to polymer formation. Therefore, increasing H_2_ in the Ar:H_2_ mixture generally created more polymer-promoting conditions in the process chamber, explaining the decreased etch rates and increased etch selectivity shown in Fig. 2a.

When C_4_F_8_ was increased in the CF_4_:C_4_F_8_ gas mixture while maintaining Ar:H_2_ at 1:3 (Fig. 2e,f), high C/F ratio radicals such as CF, C_2_F_4_, and C_3_F_5_ increased while low C/F ratio radicals such as CF_3_ decreased. Notably, C_2_F_4_ and C_3_F_5_ (which are primarily dissociated from C_4_F_8_) were absent when using pure CF_4_. Despite maintaining the same Ar:H_2_ ratio, we observed decreased H and H_2_ without Ar variation, accompanied by HF formation. These results indicate that increasing C_4_F_8_ created even more polymer-promoting conditions than increasing H_2_ in the Ar:H_2_ mixture, consistent with the observed decreased etch rates and significantly enhanced etch selectivity.

**Fig. 3:**
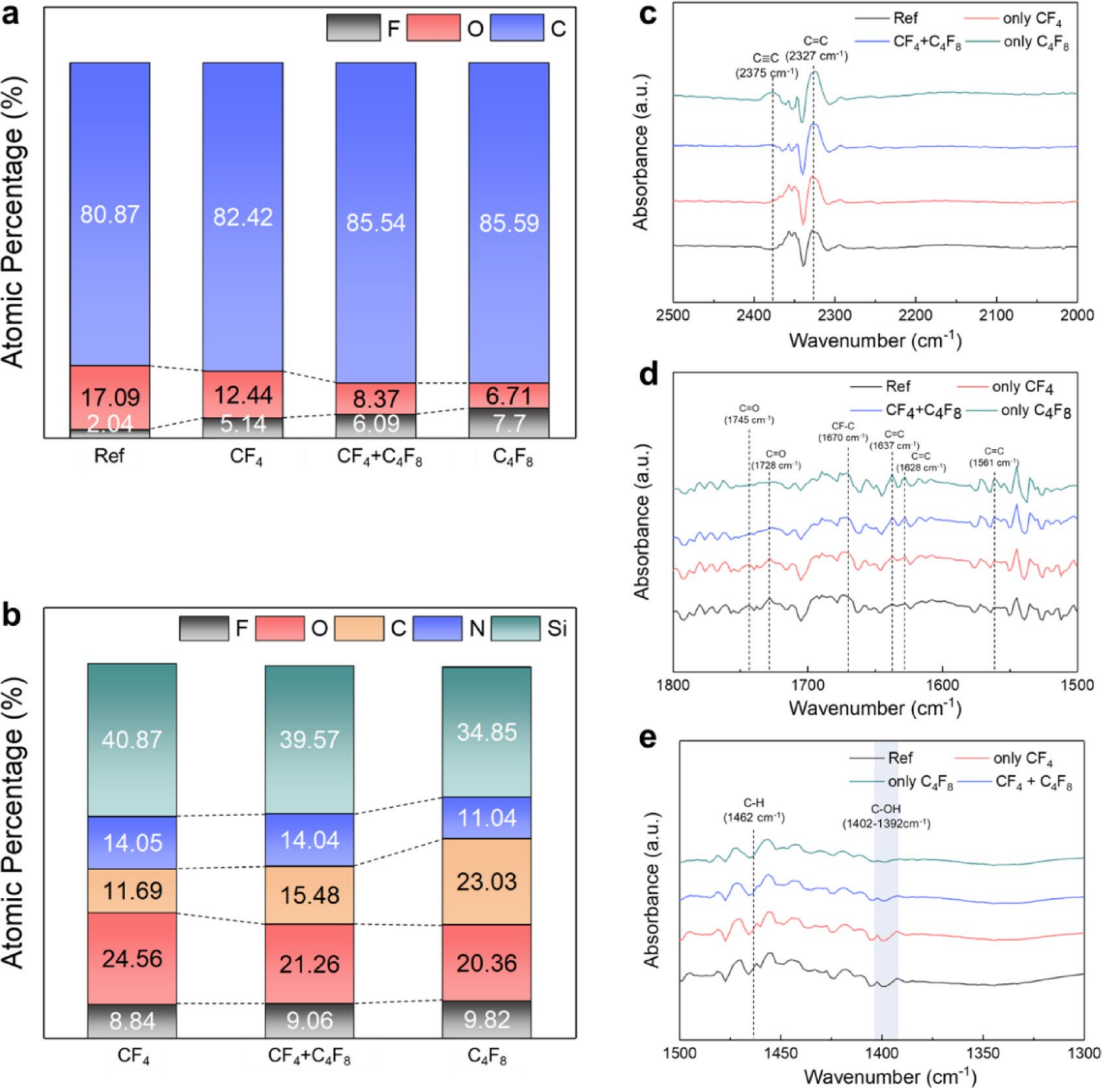
Surface analysis of EUV PR and SiON for continuous DC grid mode. XPS analysis showing atomic composition (%) of: (**a**) EUV PR surfaces before etching (Ref) and after etching using CF_4_ only, CF_4_:C_4_F_8_ (1:1), and C_4_F_8_ only (showing only F, O, and C which are the major components). (**b**) SiON surfaces after etching using the same gas conditions (showing F, O, C, N, and Si components), with Ar:H_2_ ratio of 1:3 in all cases. FTIR spectra of EUV PR comparing reference and etched samples: (**c**) 2000 ~ 2500 cm^−1^ range showing C$\equiv$C (2375 cm^−1^) and C = C (2327 cm^−1^) stretching vibrations; (**d**) 1500 ~ 1800 cm^−1^ range showing C=O (1745 cm^−1^), CF-C (1670 cm^−1^), and other C=C stretching vibrations; (**e**) 1300 ~ 1500 cm^−1^ range showing C-H (1462 cm^−1^) and C-OH vibrations.

The atomic compositions on the surfaces of EUV PR and SiON after etching using CF_4_ only, CF_4_:C_4_F_8_ = 1:1, and C_4_F_8_ only (fed to the process chamber) while keeping Ar:H_2_ at 1:3 were measured by X-ray Photoelectron Spectroscopy (XPS). The results are shown in Figs. 3a and b, respectively. The etch time was 3 min, and other process conditions matched those in Fig. 2b. For EUV PR, the atomic composition before the etchingwas included for comparison, showing only the major components (C, F, and O).

As shown in Fig. 3a, even though F is included in the reference EUV PR, the increasing C_4_F_8_ ratio elevated the F percentage on the EUV PR surface during etching, indicating enhanced fluorocarbon polymer layer formation. Figure 3b shows that increasing the C_4_F_8_ ratio led to higher C and F concentrations with decreased Si, O, and N, further confirming the formation and increased thickness of a fluorocarbon layer on the etched SiON surface. (The binding states of carbon on the EUV PR and the binding states of carbon and silicon on the etched SiON surface are shown in Supplementary Figs. S4 and S5, respectively.) These fluorocarbon layers, related to the changing radical composition in the process chamber, are responsible for the lower SiON etch rates and increased etch selectivity.

The EUV PR surfaces before and after etching (conditions in Fig. 3a) were also analyzed using Fourier Transform Infrared Spectroscopy (FTIR). The results are shown in Fig. 3c–e for different wavenumber ranges: (c) 2000–2500 cm^−1^, (d) 1500–1800 cm^−1^, and (e) 1300–1500 cm^−1^ (complete FTIR spectra in Supplementary Fig. S6). Compared to the reference, samples treated from pure CF_4_ to pure C_4_F_8_ showed increased intensities of C=C stretching vibration (1628, 1637, 2327 cm^−1^)^28–30^, C=C stretching vibration of benzene ring (1561 cm^−1^)^31^, C$\equiv$C vibration (2375 cm^−1^)^32^ peaks. Simultaneously, these samples showed decreased intensities of C=O stretching vibration (1728, 1745 cm^−1^)^33,34^ and C-H bending vibration (1462 cm^−1^)^35^ peaks. These results indicate that higher C_4_F_8_ ratios increased carbon-to-carbon binding states, particularly double and triple bonds. The increase in carbon-to-carbon bonding states on the EUV PR during etching may involve the formation of triple bonds, which have higher bond energies and shorter bond lengths than single or double bonds^36^. These changes in bonding characteristics may lead to stress generation within the EUV PR. Therefore, the increased LER observed with increasing C_4_F_8_ ratio is believed to be related to the higher carbon-to-carbon bonding states on the etched EUV PR surface.

### Ion beam etching with the pulsed DC grid mode

To improve etch selectivity without increasing LER, a grid pulsing technique was investigated, illustrated in Fig. 1b. In this study, the Ar:H_2_ ratio was maintained at 1:3 and the CF_4_:C_4_F_8_ ratio at 1:1, while varying the grid pulse duty ratio from 20 to 100% (continuous) at a grid pulse frequency of 100 Hz.

**Fig. 4:**
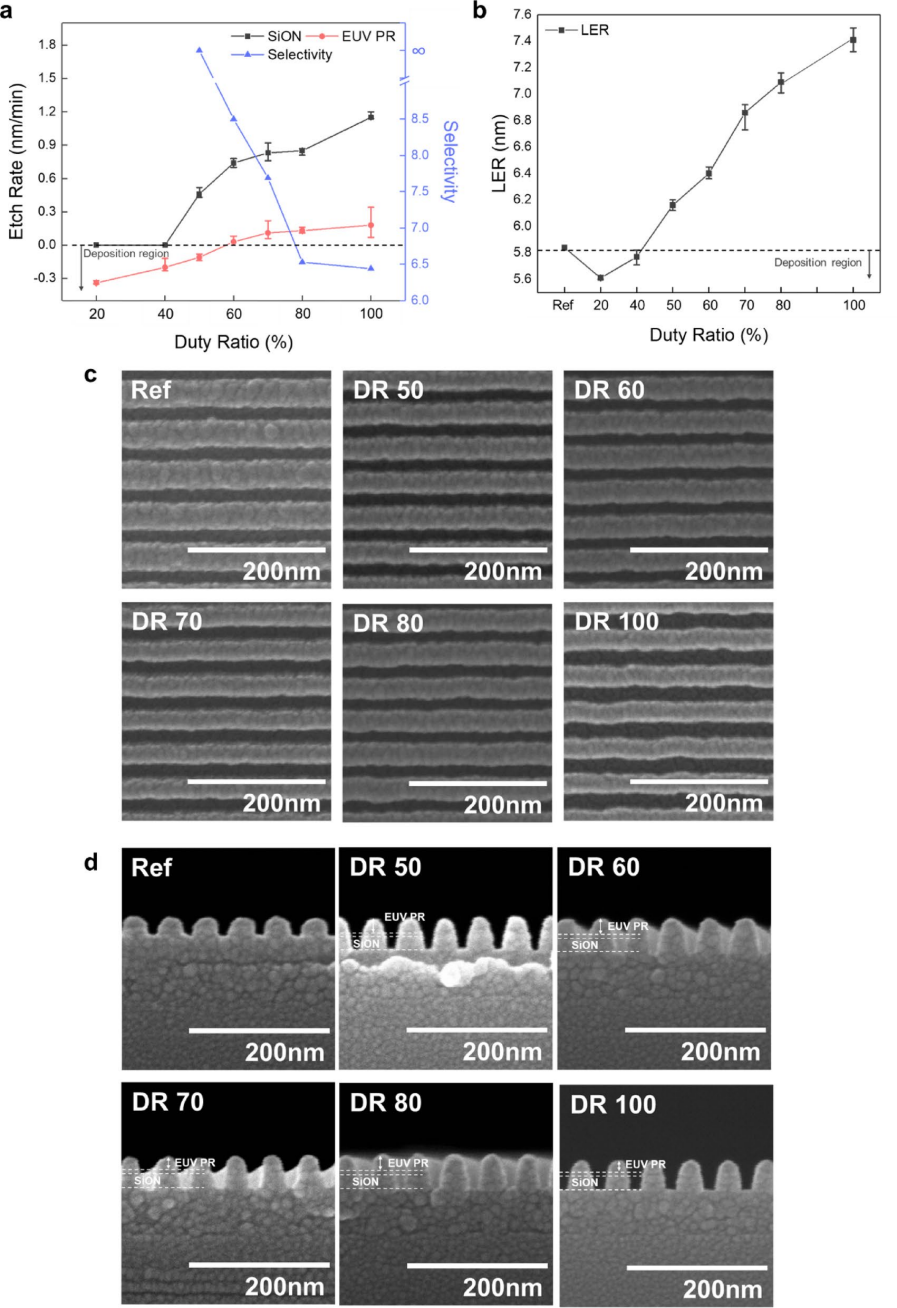
Etch characteristics for pulsed DC grid mode. (**a**) Etch rates of SiON and EUV PR and their selectivity ratios as a function of grid pulse duty ratio (20–100%), showing deposition region at lower duty ratios. (**b**) Line edge roughness (LER) of EUV PR measured after etching 20-nm-thick SiON with different pulse duty ratios, including reference (Ref) value. (**c**) Top-view SEM images showing line patterns for reference and after etching with duty ratios of 50–100% (200 nm scale bars). (**d**) Cross-sectional SEM images showing the EUV PR/SiON interface for the same conditions (200 nm scale bars).

As shown in Fig. 4a, decreasing the duty ratio from 100 to 50% reduced the etch rates of SiON (from 1.15 to 0.46 nm/min) and EUV PR (from 0.18 to − 0.11 nm/min, indicating deposition rather etching). Consequently, the etch selectivity improved from 6.5 to ∞. Further reduction of the grid pulse duty ratio to 20% increased deposition on the EUV PR without etching the SiON.

Following the complete etching of the 20-nm-thick SiON layer, LER of the EUV PR was measured using LACERM software across different grid pulse duty ratios (20 to 100%), with results shown in Fig. 4b. The LER value for reference EUV PR before etching was also included for comparison. By decreasing the pulse duty ratio from 100 to 50%, we observed a reduction in LER from 7.4 to 6.1 nm (approaching the reference value of 5.8 nm). Further reduction of the duty ratio from 40 to 20% decreased the LER below the reference value, though this was attributed to fluorocarbon layer deposition on the EUV PR surface.

Figure 4c shows top-view SEM images of the EUV PR for duty ratios ranging from 50 to 100%, while Fig. 4d presents the corresponding cross-sectional images. (SEM images for pulse duty ratios of 40% and 20%, where no SiON etching occurred, are available in Supplementary Fig. S7). Both top and cross-sectional SEM images confirm decreased LER and thicker remaining EUV PR with decreasing pulse duty ratio, demonstrating enhanced etch selectivity of SiON over EUV PR after 20 nm SiON etching.

### Etch mechanism analysis in the pulsed DC grid mode

To understand the etch characteristics observed in the pulsed duty mode, the dissociated gas species in the process chamber were investigated using QMS. Results are presented in the Supplementary Fig. S8. Despite changing the pulse duty ratio from 100 to 20% while maintaining the Ar:H_2_ ratio at 1:3 and CF_4_:C_4_F_8_ ratio at 1:1, no significant variations were observed in the intensity ratios among the dissociated species (C_x_F_y_, H_x_, and Ar) in the process chamber. This indicates that the pulsing had minimal effect on the relative proportions of dissociated gas species.

**Fig. 5:**
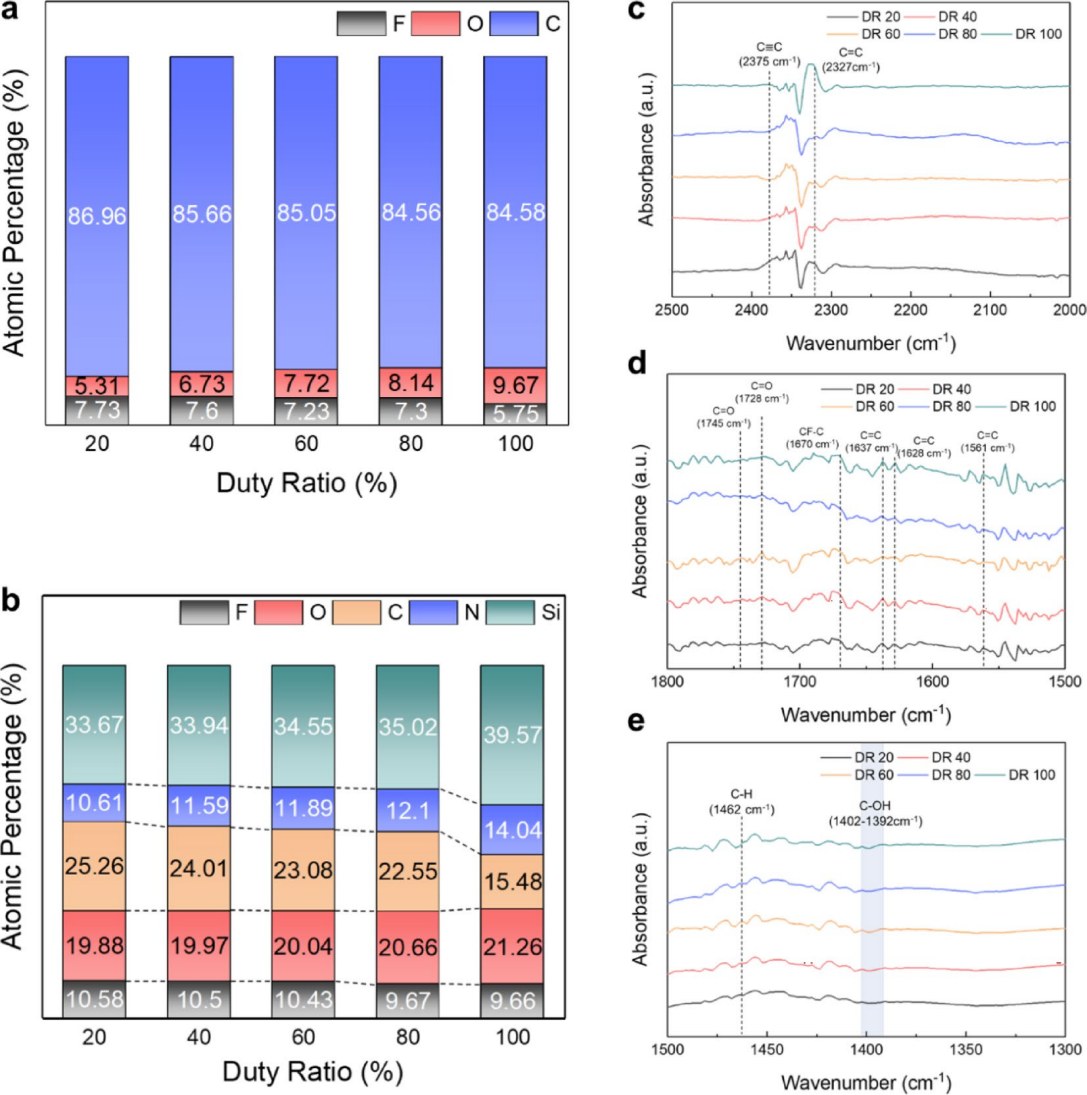
Surface analysis of EUV PR and SiON for pulsed DC grid mode. XPS analysis showing atomic composition (%) of: (**a**) EUV PR surfaces and (**b**) SiON surfaces after etching with different pulse duty ratios (20%–100%), with Ar:H_2_ = 1:3 and CF_4_:C_4_F_8_ = 1:1. FTIR spectra of EUV PR showing bonding state changes across duty ratios in wavenumber ranges: (**c**) 2000–2500 cm^−1^ with C$\equiv$C (2375 cm^−1^) and C=C (2327 cm^−1^) stretching vibrations; (**d**) 1500–1800 cm^−1^ with C=O (1728, 1745 cm^−1^) and various C=C vibrations; (**e**) 1300–1500 cm^−1^ with C-H (1462 cm^−1^) and C-OH vibrations.

The surfaces of EUV PR and SiON after 3 min of etching under the conditions described in Fig. 4a were analyzed using XPS. Figure 5a,b, show the atomic percentages of major species on EUV PR and SiON surfaces, respectively. As shown in Fig. 5a, decreasing the pulse duty ratio from 100 (continuous) to 20% increased the fluorine percentage from 5.75 to 7.73% with carbon percentage from 84.58 to 86.96% on the EUV PR surface. This indicates enhanced fluorocarbon layer formation with a decreasing duty ratio, which contributes to decreased EUV PR etch rates. This decrease is caused by reduced ion bombardment time at lower duty ratios, ultimately leading to deposition rather than etching at duty ratios ≤  40%.

The SiON surfaces exhibited a similar trend, as shown in Fig. 5b. As the duty decreased, increased C (from 15.48 to 25.26%) and F (from 9.66 to 10.58%) concentrations, with corresponding decreases in Si (from 39.57 to 33.67%), O (from 21.26 to 19.88%), and N (from 14.04 to 10.61%) were observed. These changes correlate with the decreased SiON etch rate at lower duty ratios and the cessation of etching at duty ratios ≤ 40%. (The binding states of carbon on the EUV PR and the binding states of carbon and silicon on the etched SiON surface are presented in Supplementary Figs. S9 and S10, respectively.)

To elucidate the changes in LER with varying pulse duty ratios, the etched EUV PR was analyzed using FTIR. Results are shown in Fig. 5c–e for different wavenumber ranges: (c) 2000–2500 cm^−1^, (d) 1500–1800 cm^−1^, and (e) 1300–1500 cm^−1^. (Complete FTIR spectra are available in Supplementary Fig. S11).

Similar to the continuous DC modes in Fig. 3c-e, we observed peaks associated with C=C stretching vibration (1628, 1637, 2327 cm^−1^), C=C stretching vibration of the benzene ring (1561 cm^−1^), C$\equiv$C vibration (2375 cm^−1^) peaks, C=O stretching vibration (1728, 1745 cm^−1^), and C-H bending vibration (1462 cm^−1^). As the pulse duty ratio decreased from 100 to 60%, peak intensities related to higher carbon-to-carbon binding states (C=C stretching, benzene ring C=C stretching, and C$\equiv$C vibration) decreased, while C = O stretching vibration and C-H bending vibration peaks increased. This indicates lower stress on the EUV PR with a decreasing pulse duty ratio in this range.

For EUV PR etched at duty ratios of 40% and 20%, different behavior were observed: decreases in both C=C/C$\equiv$C vibration peaks and C=O stretching/C-H bending vibration peaks with decreasing duty ratio. This difference is attributed to fluorocarbon layer deposition on the EUV PR surface. These results suggest that the decreased LER observed with a decreasing grid duty ratio relates to reduced deformation of the EUV PR during etching.

**Fig. 6:**
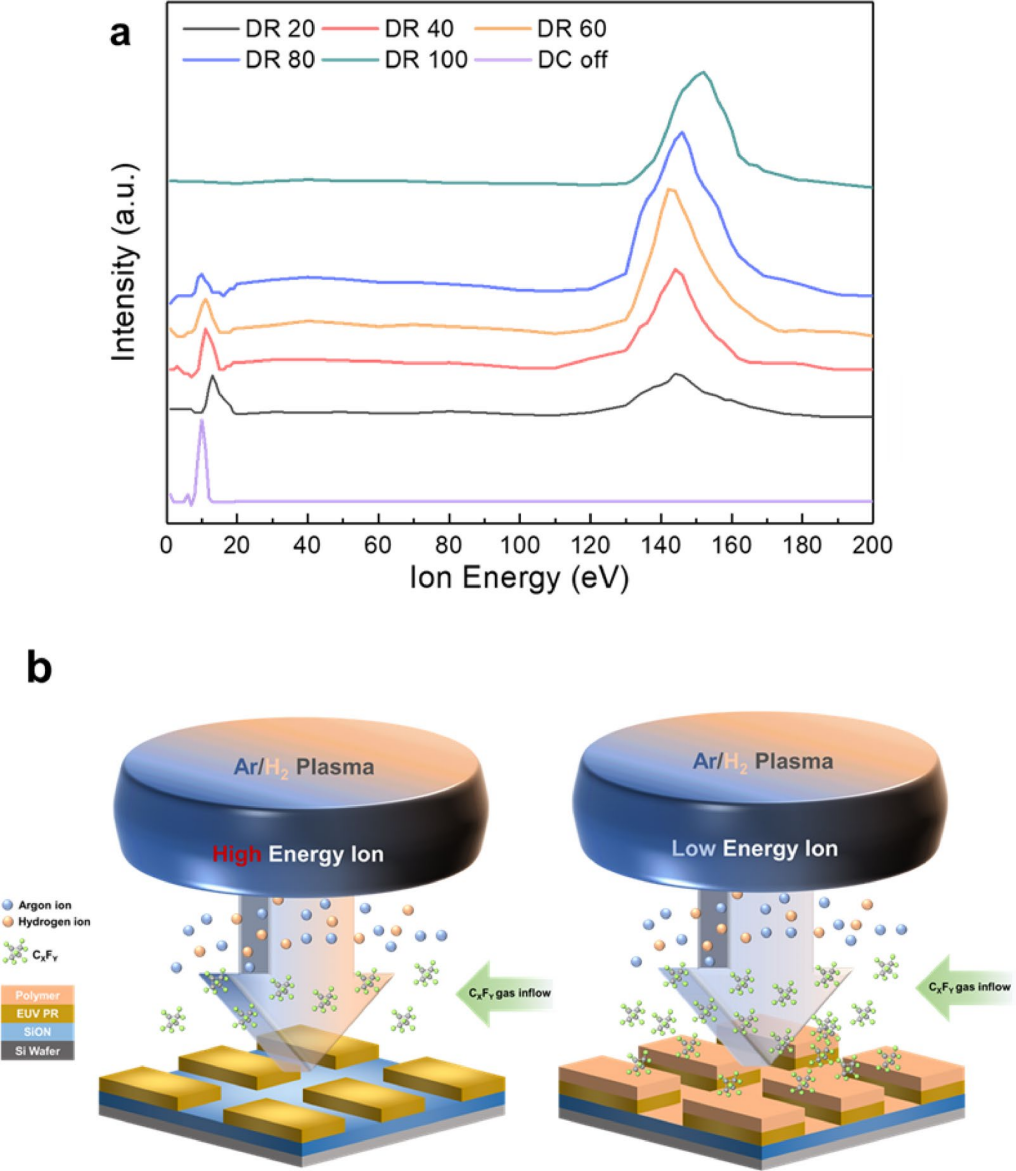
Ion energy analysis and etching mechanism. (**a**) Time-average ion energy distribution measurements at the substrate for different grid pulse duty ratios (DR 20–100%) and DC off condition, showing the transition from low-energy (~ 10–20 eV) to high-energy (~ 150–160 eV) ion bombardment. (**b**) Schematic illustration of the etching mechanism showing two modes: pulse-on period with high-energy ion bombardment leading to etching (left), and pulse-off period with low-energy ion bombardment promoting polymer formation (right). The diagram shows the Ar/H_2_ plasma source, ion trajectories, C_x_F_y_ gas inflow, and the effects on the patterned substrate.

For the pulsed DC mode, conditions in Fig. 4a, the time-averaged energy distribution of ions bombarding the substrate was measured using a retarding grid ion energy analyzer. Figure 6a shows the results for pulse duty ratios ranging from 20 to 100%. The ion energy distribution without applied grid voltages (0 V to all grids) was also included to represent the grid pulse-off period. (Ion beam currents as a function of ion energy analyzer voltage are shown in Supplementary Fig. S12).

During the pulse-off period, low ion bombardment energy (~ 10 eV) was observed resulting from the plasma potential of the ICP source. In contrast, at 100% duty ratio (continuous DC mode), only high ion bombardment energy approaching the 1st grid voltage (+ 150 V) + plasma potential (~ 10 eV) was observed. As the duty ratio decreased, the high-ion-energy peak intensity (pulse-on period) diminished while the low-ion-energy peak intensity (pulse-off period) increased. The high-energy peak position appeared slightly reduced after pulsing, possibly due to challenges in ion acceleration between the 1st and 2nd grids during rapid voltage transitions (0 to + 150 V) at 100 Hz.

Based on these ion energy distributions, the etching mechanism of the grid pulsing technique could be elucidated, illustrated schematically in Fig. 6b. The process alternates between pulse-on and pulse-off periods. During the pulse-on period, high-energy Ar^+^/H_x_^+^ ion beams dissociate the fluorocarbon gases (C_4_F_8_ + CF_4_) in the process chamber are etch the substrate through ion bombardment. During the pulse-off period, the low-energy ion beam promotes fluorocarbon polymer layer formation through polymerization without significant etching.

The fluorocarbon polymer layer thickness increases with decreasing pulse duty ratio. However, the reduced ion bombardment at lower duty ratios causes less deformation of the EUV PR, resulting in lower LER while simultaneously decreasing etch rates and increasing selectivity. When the pulse-off time becomes excessive (duty ratios ≤ 40%), the fluorocarbon deposition rate exceeds the etching rate during the pulse-on period, resulting in either no etching or net deposition on both EUV PR and SiON surfaces.

## Conclusion

In this study, the etching characteristics of EUV PR and SiON underlayers were investigated using an ion beam operated in both continuous DC mode and pulsed DC mode. We fed a mixture of Ar and H_2_ to the ion beam source while introducing a fluorocarbon gas mixture (CF_4_ and C_4_F_8_) into the process chamber. In the continuous DC mode, the etch selectivity of SiON over EUV PR increased while the SiON etch rate decreased with both increasing H_2_ ratio in the ion beam source and increasing C_4_F_8_ ratio in the process chamber. Notably, although increasing the C_4_F_8_ ratio in CF_4_ + C_4_F_8_ improved etch selectivity, it also increased LER due to enhanced carbon-to-carbon bonding in the etched EUV PR.

To improve these etching characteristics, we implemented grid voltage pulsing. By decreasing the grid pulse duty ratio from 100% (continuous) to 50%, we observed decreased SiON etch rates accompanied by significantly increased etch selectivity of SiON over EUV PR with improved LER. This enhancement resulted from increased fluorocarbon layer deposition during the pulse-off period, when low-energy Ar^+^/H_x_^+^ ion bombardment (~ 10 eV) promoted polymer formation without aggressive etching, alternating with substrate etching by high-energy Ar^+^/H_x_^+^ ion bombardment (~ 160 eV) during the pulse-on period. However, excessive pulse-off periods (duty ratios ≤ 40%) caused fluorocarbon deposition to exceed etching capacity, resulting in either no etching or net deposition on both EUV PR and SiON surfaces.

Using the optimized grid pulsing mode with Ar:H_2_ ratio = 1:3 to the ion beam source and CF_4_:C_4_F_8_ = 1:1 to the process chamber, we achieved an etch selectivity of SiON over EUV PR approaching ∞ at 50% duty ratio. This process significantly improved LER from ~ 9.6 nm with C_4_F_8_-only processes to ~ 7.4 nm under optimized gas conditions, and further to ~ 6.1 nm with grid pulsing (∆LER = 0.3 nm compared to the reference). This represents a ~ 20% improvement compared to the optimized continuous gas conditions. It is believed that the grid pulsing ion beam etching method can be applied to various next-generation hard mask etching processes using EUV PR patterns where high etch selectivity is required without LER degradation.

## Methods

### Grid pulsed ion beam etch system

An inductively coupled plasma (ICP) type source was used, as shown in Fig. 1a, with plasma discharge generated by applying a 13.56 MHz radio frequency (RF) power of 300 W to the ICP coil. We injected an Ar/H_2_ gas mixture into the ion beam source chamber (top) and a C_x_F_y_ gas mixture into the process chamber (bottom) without plasma generation. This approach aimed to reduce the rapid decrease in etch rate due to excessive polymer generation by plasmas and to minimize grid and chamber contamination issues.

For extracting reactive ions from the ion source, a three-graphite-grid assembly was employed. A positive voltage was applied to the 1st grid facing the plasma for ion energy control, a negative voltage to the 2nd grid to control the flux of extracted ions, while the 3rd grid facing the process chamber was grounded. The substrate temperature was maintained at room temperature (20 °C) using a chiller, and the chamber base pressure was kept below 5 × 10^−6^ Torr using a turbo molecular pump before the process. For grid pulsing, pulsed DC power supplies were connected to both the 1st and 2nd grids instead of conventional DC power supplies. Figure 1b shows the 1st and 2nd grid voltages for both conventional DC mode and pulsed DC mode. During pulsed grid operation, we varied the pulse duty-on percentage while continuously operating the ion beam source plasma. The grid pulsing frequency was fixed at 100 Hz.

### EUV PR pattern condition

The EUV PR pattern, shown in Fig. 1c, consisted of a line pattern with a 30 nm line width. The pattern structure included a ~ 30 nm thick chemically amplified resist (CAR) type EUV PR as the mask layer, with a bottom anti-reflective carbon (BARC) layer, SiON, and a spin-on-carbon (SOC) layer deposited sequentially on the Si wafer beneath. This pattern was then etched as shown in Fig. 1d for evaluation and analysis.

### SiON etch process conditions

For the SiON etch process, we injected an Ar/H_2_ gas mixture at a pressure of 2 mTorr into the top ion beam source for discharge, with gas ratios ranging from 4:1 to 1:4. At the bottom of the process chamber, a C_x_F_y_ gas mixture was injected at a pressure of 0.5 mTorr (CF_4_:C_4_F_8_ gas ratio varied from 1:0 to 0:1), maintaining a total process chamber of 2.5 mTorr. We applied a voltage of + 150 V was applied to the 1st grid and 200 V to the 2nd grid. For the grid pulsing mode, gas pressure, RF power, and DC voltage were kept constant, while the grid pulse duty-on ratio (DR) was varied from 20 to 100% (continuous mode) at a pulse frequency of 100 Hz.

### Analytical methods and instruments

After etching, we examined changes in etch depths and profiles using a field emission scanning electron microscope (FE-SEM; Hitachi, S-4700). Line edge roughness (LER) was measured using a MATLAB-based software called line and contact edge roughness meter (LACERM).

To analyze gas decomposition during processing, a quadrupole mass spectrometer (QMS; SRS, RGA 300) was employed. The QMS operated in residual gas analyzer mode, where gases extracted from the process chamber were ionized and partially fragmented with 70 eV electrons before analysis.

X-ray photoelectron spectroscopy (XPS; Thermo VG, MultiLab 2000) with an Al Kα source was used to analyze the chemical bonding states and atomic composition of the EUV PR surface before and after etching. Binding energies were calibrated using the C 1 s peak as a reference. Peak fitting was performed using Thermo Avantage software, applying a Shirley background.

The changes in bonding formed on the EUV PR surfaces were analyzed using Fourier transform infrared spectroscopy (FTIR; Shimadzu, IRTracer-100). The time-averaged ion energy distribution to the wafer during processing was analyzed using a retarding grid ion energy analyzer positioned on the substrate.

## Electronic supplementary material

Below is the link to the electronic supplementary material.

Supplementary Material 1

## Data Availability

Data will be made available on request to Geun Young Yeom. (gyyeom@skku.edu).
