# Supplementary material for: Enhanced etch characteristics of EUV PR masked SiON through the ion beam grid pulsing technique
Source: Sci Rep. 2025 Jun 6;15:19920. doi: 10.1038/s41598-025-04632-x (PMC12144267; doi:10.1038/s41598-025-04632-x)
Supplement: Supplementary file 1 — Supplementary Material 1 [file 41598_2025_4632_MOESM1_ESM.docx]

**Supplementary information**

**Enhanced Etch Characteristics of EUV PR Masked SiON through Ion Beam Grid Pulsing Technique**

Hae In Kwon^1,=^, Yun Jong Jang^1,=^, Kyoung Chan Kim^1^, Hong Seong Gil^1^, Ju Young Kim^3^, Seong Hyun Ryu^5^, Do Seong Pyun^4^, Dae Whan Kim^4^, Woo Chang Park^1^, Ji Yeon Lee^4^, Jin Woo Park^6^,Sang Wuk Park^6^ and Geun Young Yeom^1,2,4,*^

^1^School of Advanced Materials Science and Engineering, Sungkyunkwan University, Suwon 16419, Republic of Korea

^2^SKKU Advanced Institute of Nano Technology (SAINT), Sungkyunkwan University, Suwon 16419, Republic of Korea

^3^Department of Photovoltaic System Engineering, Sungkyunkwan University, Suwon 16419, Republic of Korea

^4^Department of Semiconductor Display Engineering, Sungkyunkwan University, Suwon 16419, Republic of Korea

^5^School of Chemical Engineering, Sungkyunkwan University, Suwon 16419, Republic of Korea

^6^Advanced Process Development, Semiconductor R&D Center, Samsung Electronics Co. Ltd., Hwaseong 18448, Republic of Korea

=These authors contributed equally to this work.

*E-mail: [gyyeom@skku.edu](mailto:gyyeom@skku.edu)

**
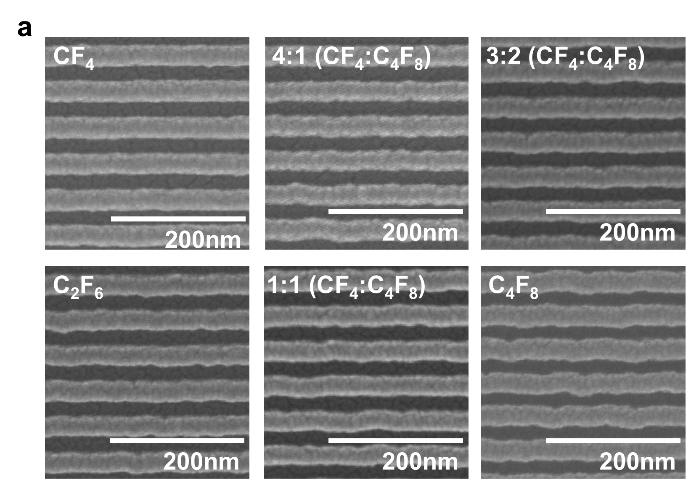

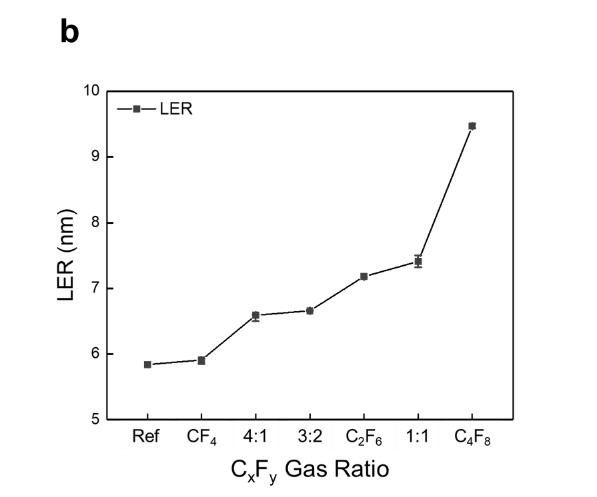
**

**Figure S1.** Surface analysis after etching according to the C_x_F_y_ ratio. **a**, Top SEM images and **b**, Line-edge roughness of EUV PR by LACERM before and after etching 20 nm thick SiON with different ratios of CF_4_:C_4_F_8_ and C_2_F_6_ while keeping the Ar:H_2_ ratio at 1:3 in conditions of Figure 2b.

Top SEM images of EUV PR after the etching 20 nm thick SiON with C_x_F_y_ ratios while keeping the Ar:H_2_ ratio at 1:3 in conditions of Figure 2b. The LER measured by LACERM showed the increased LER compared to the reference.


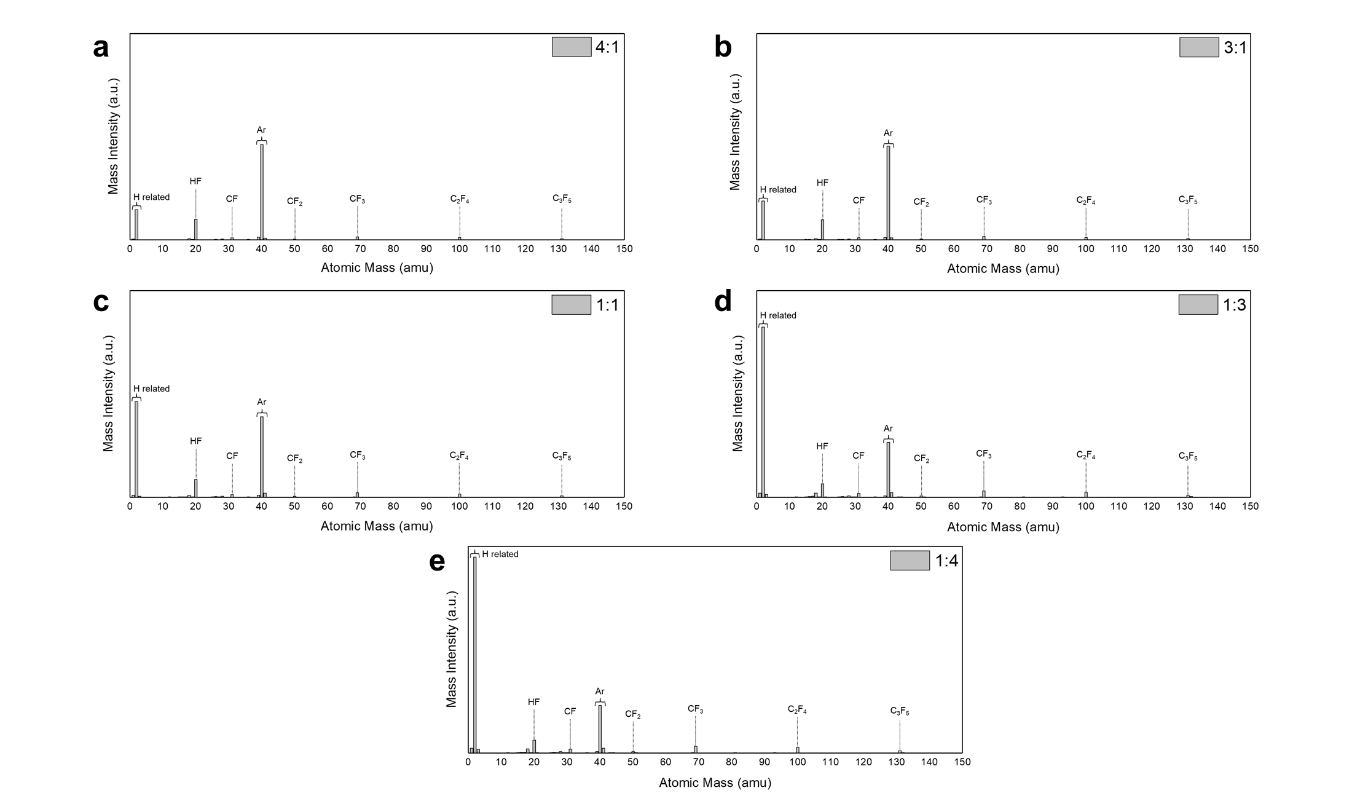


**Figure S2.** QMS data in the process chamber during etching for the different Ar:H_2_ gas ratios in Fig. 2a. **a**, 4:1, **b**, 3:1, **c**, 1:1, **d**, 1:3, and **e**, 1:4 of Ar:H_2_ while keeping the ratio of CF_4_:C_4_F_8_ at 1:1.

The increase of H_2_ in the Ar:H_2_ gas mixture increased H and H_2_ while decreasing Ar due to the increase of H_2_ in the gas mixture. In addition, the increase of CF_x_ (x=1~3) and C_x_F_y_ such as C_2_F_4_ and C_3_F_5_ dissociated from CF_4_ and C_4_F_8_ and the formation of HF were observed.


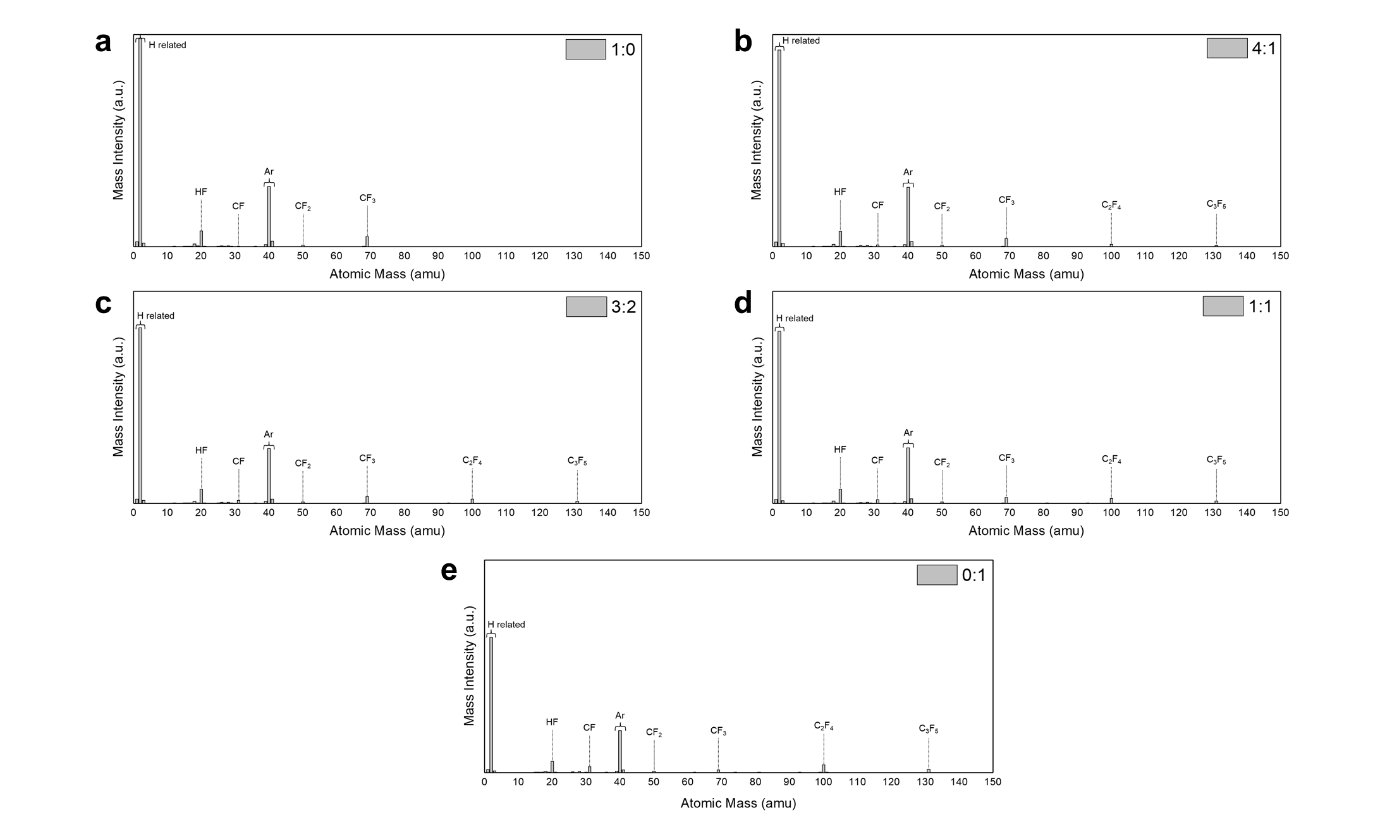


**Figure S3.** QMS data in the process chamber during etching for the different CF_4_:C_4_F_8_ gas ratios in Fig. 2b. **a**, 1:0, **b**, 4:1, **c**, 3:2, **d**, 1:1, and **e**, 0:1 while keeping the ratio of Ar:H_2_ at 1:3.

When the C_4_F_8_ was increased in CF_4_:C_4_F_8_ gas mixture while maintaining Ar:H_2_ ratio at 1:3, the high C/F ratio radicals such as CF, C_2_F_4_, and C_3_F_5_ were increased while decreasing low C/F ratio radicals such as CF_3_. In addition, no C_2_F_4_ and C_3_F_5_ which are dissociated from C_4_F_8_ were observed during using pure CF_4_.


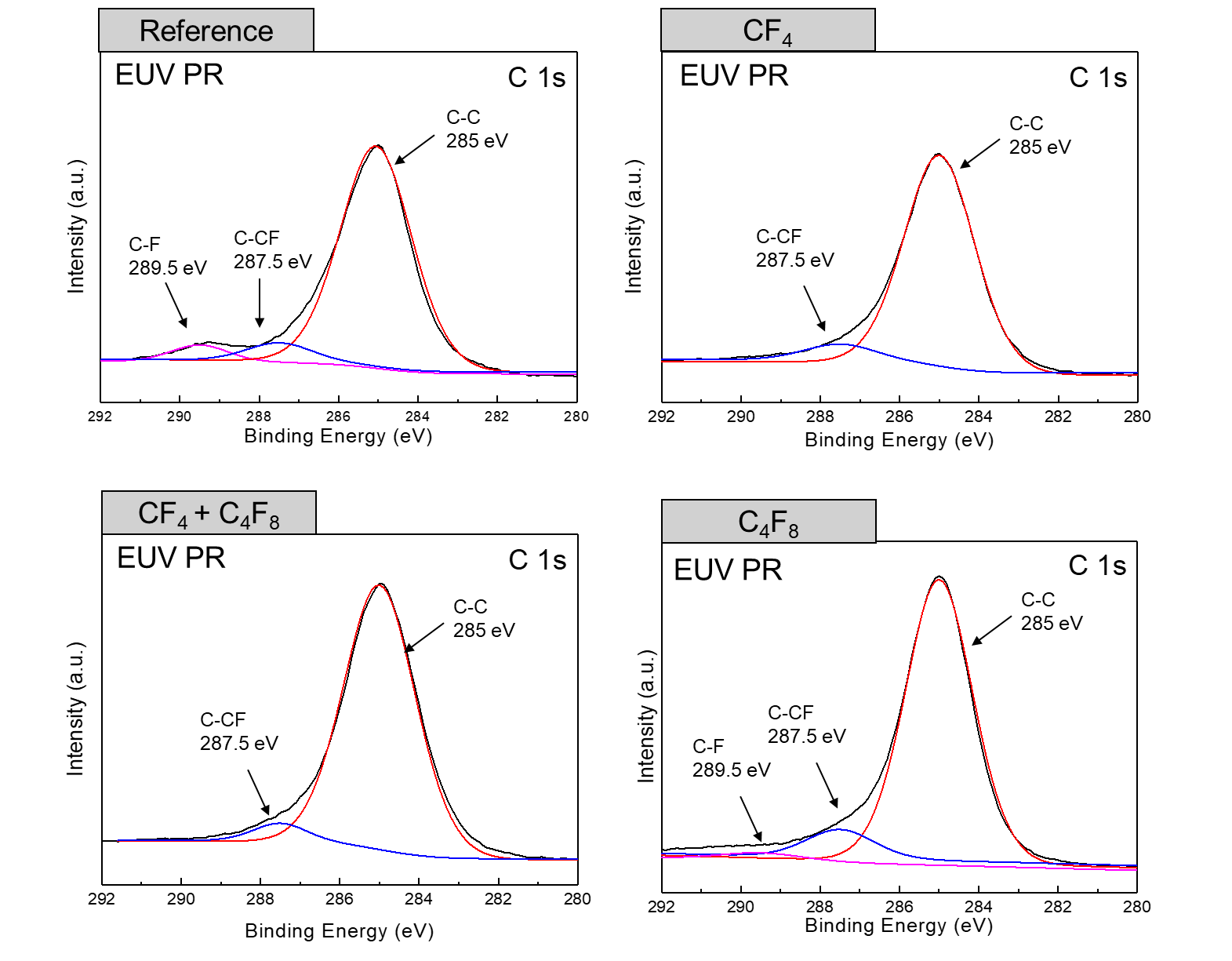


**Figure S4.** XPS C 1s binding states of EUV PR surface before and after etching using CF_4_ only, CF_4_:C_4_F_8_=1:1, and C_4_F_8_ only (fed to the process chamber) while keeping Ar:H_2_ ratio (fed to the ion beam source) at 1:3.

The binding states of carbon on the EUV surface before and after the etching using the conditions in Fig. 3a showed C-CF bonding at 287.5 eV^1,2^, C-F bonding at 289.5 eV^2,3^, in addition to C-C bonding at 285 eV^2^, and which indicates the formation of a fluorocarbon layer on the etched EUV surface.


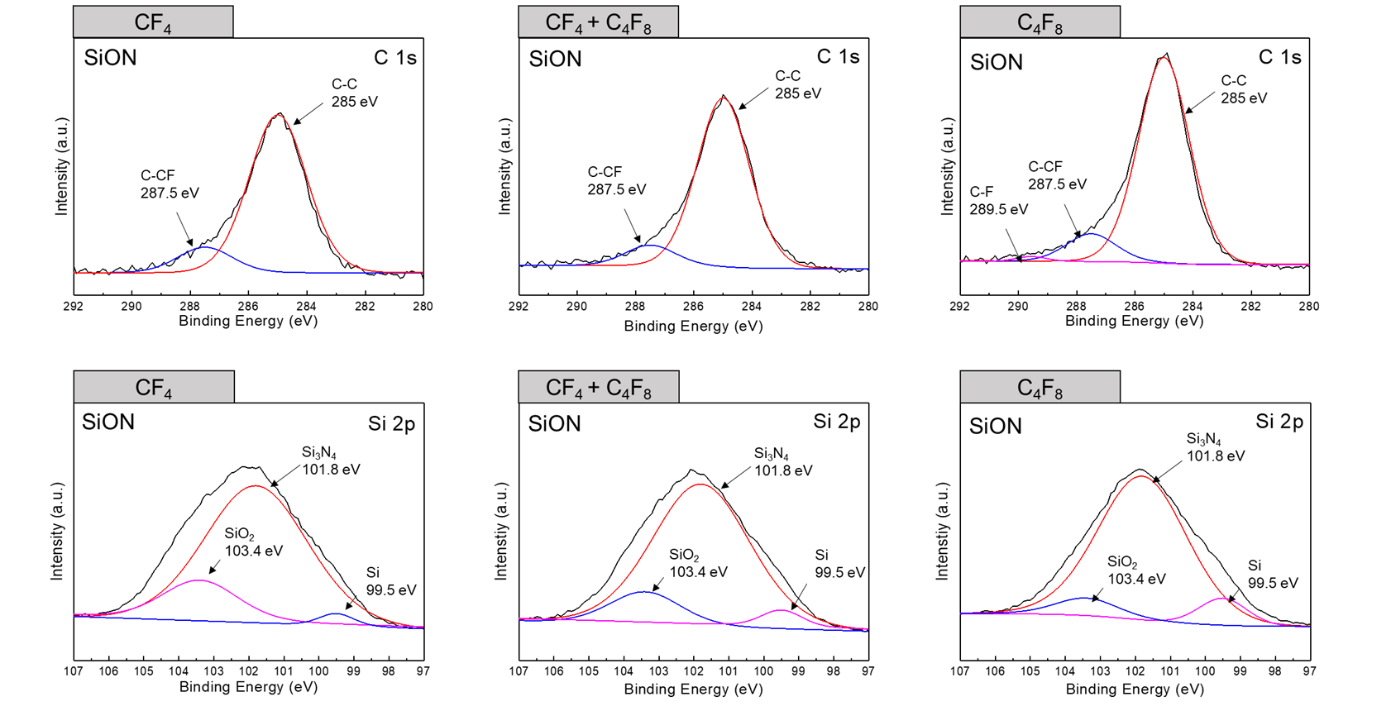


**Figure S5.** XPS C 1s and Si 2p binding states of SiON surface after etching using CF_4_ only, CF_4_:C_4_F_8_=1:1, and C_4_F_8_ only (fed to the process chamber) while keeping Ar:H_2_ ratio (fed to the ion beam source) at 1:3.

The binding states of carbon and silicon on the etched SiON surface using the conditions in Fig. 3b showed C-CF bonding in addition to C-C bonding without Si-F or Si-C bindings, which indicates the formation of a fluorocarbon polymer layer on the etched SiON surface. Additionally in Si 2p peaks, peaks related to SiO_2_ at 103.4 eV, Si_3_N_4_ at 101.8 eV in addition to Si at 99.5 eV were observed indicating the silicon oxynitride layer^4^.


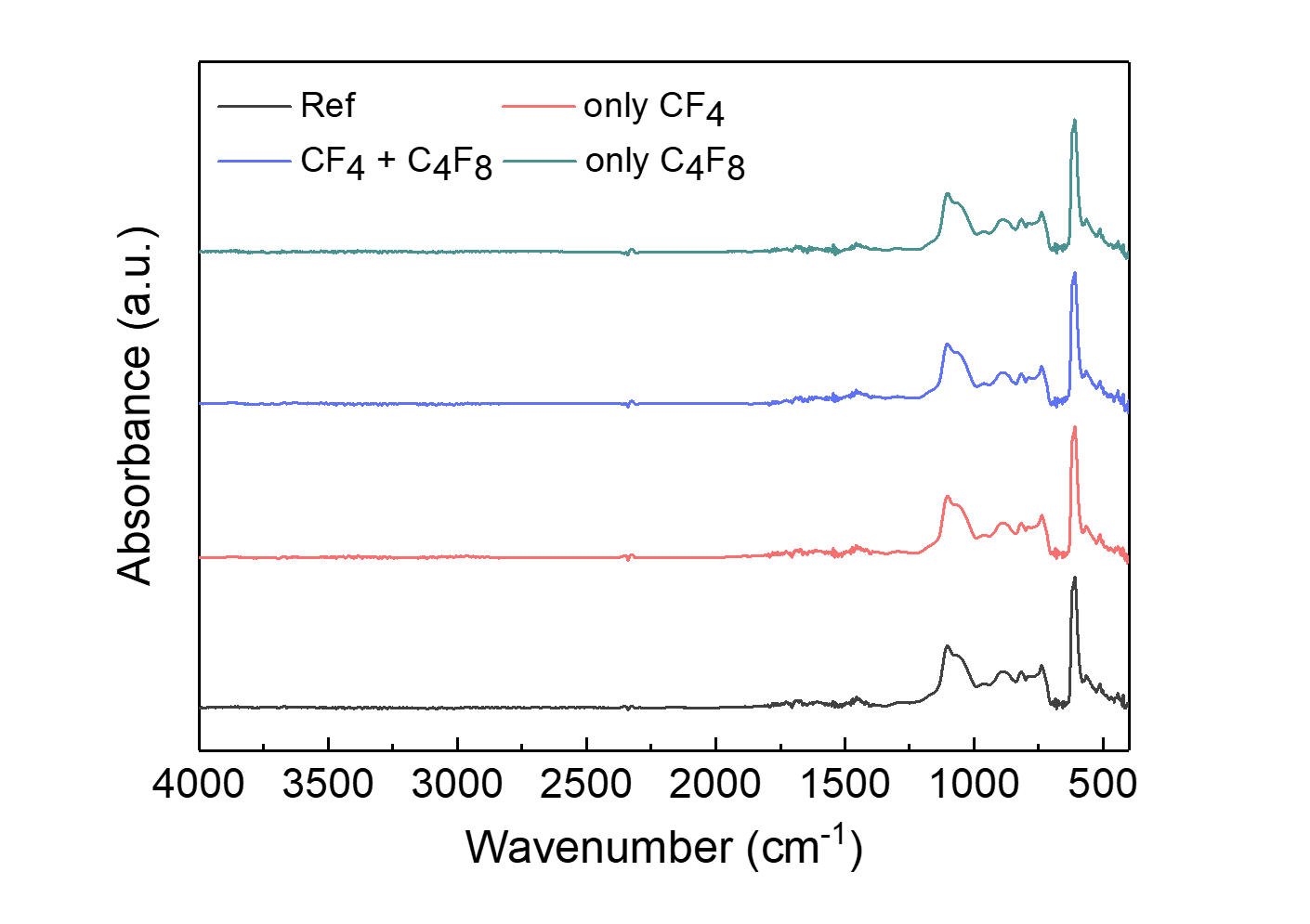


**Figure S6.** FTIR spectra of EUV PR before and after the etching using the conditions in Fig. 3a,b.

The whole FTIR spectra before and after etching appeared to show no differences but detailed spectral investigation showed the differences in binding states. Compared to the reference EUV PR, the EUV PR etched using more C_4_F_8_ in the CF_4_:C_4_F_8_ gas mixture, that is, from pure CF_4_ to pure C_4_F_8_, increased C=C stretching vibration (1628, 1637, 2327 cm^-1^)^5-7^, C=C stretching vibration of benzene ring (1561 cm-1)^8^, C$\equiv$C vibration (2375 cm^-1^)^9^ peaks while decreasing C=O stretching vibration (1728, 1745 cm^-1^)^10,11^ and C-H bending vibration (1462 cm^-1^)^12^ peaks.

**
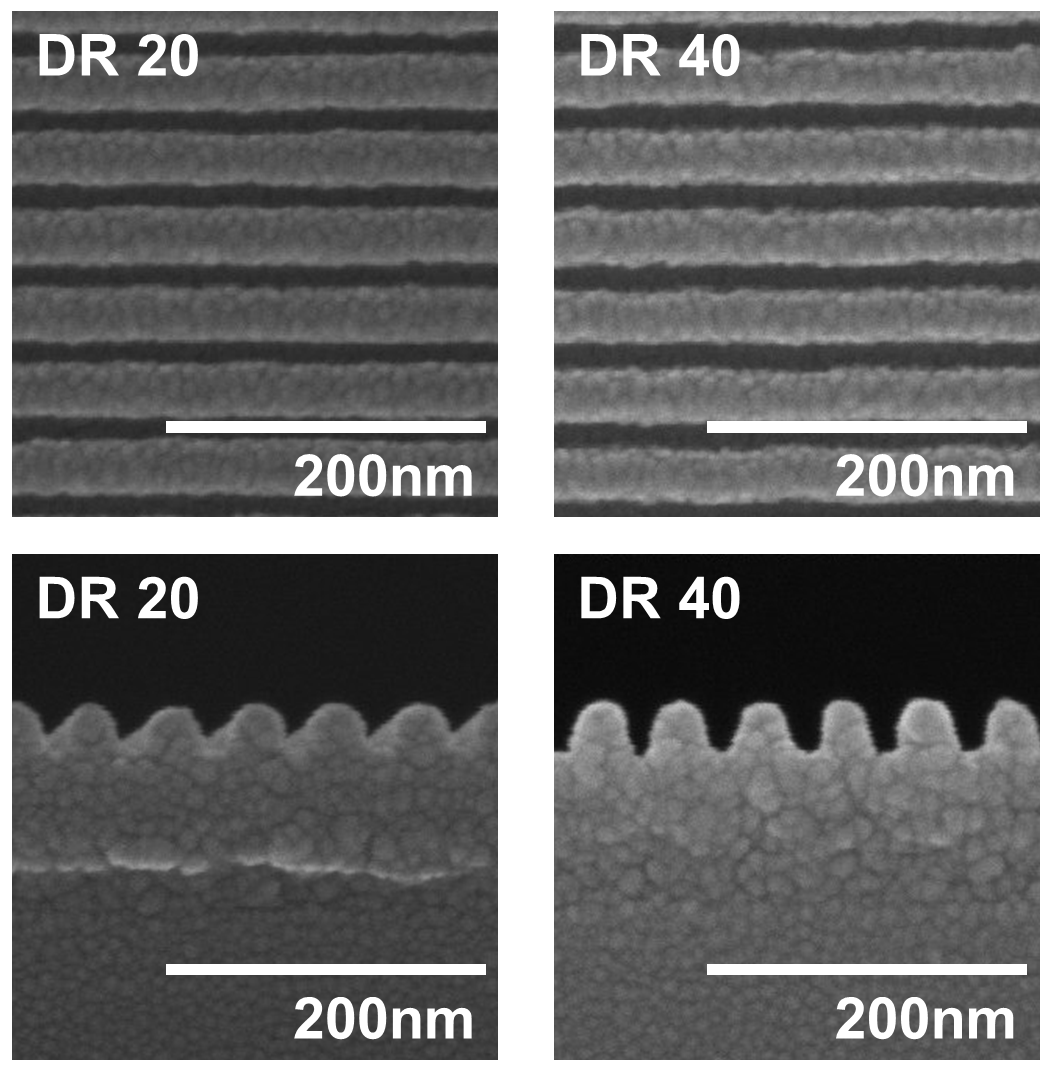
**

**Figure S7**. Surface analysis after etching according to pulse duty ratio.

Top SEM images of EUV PR after the etching 20 nm thick SiON with duty ratios of 20 and 40 % while keeping the Ar:H_2_ ratio at 1:3 and CF_4_:C_4_F_8_ ratio at 1:1 in conditions of Figure 4a. The LER measured by LACERM showed the decreased LER compared to the reference but, as shown in cross-sectional SEM images, no SiON etching was observed for these conditions.


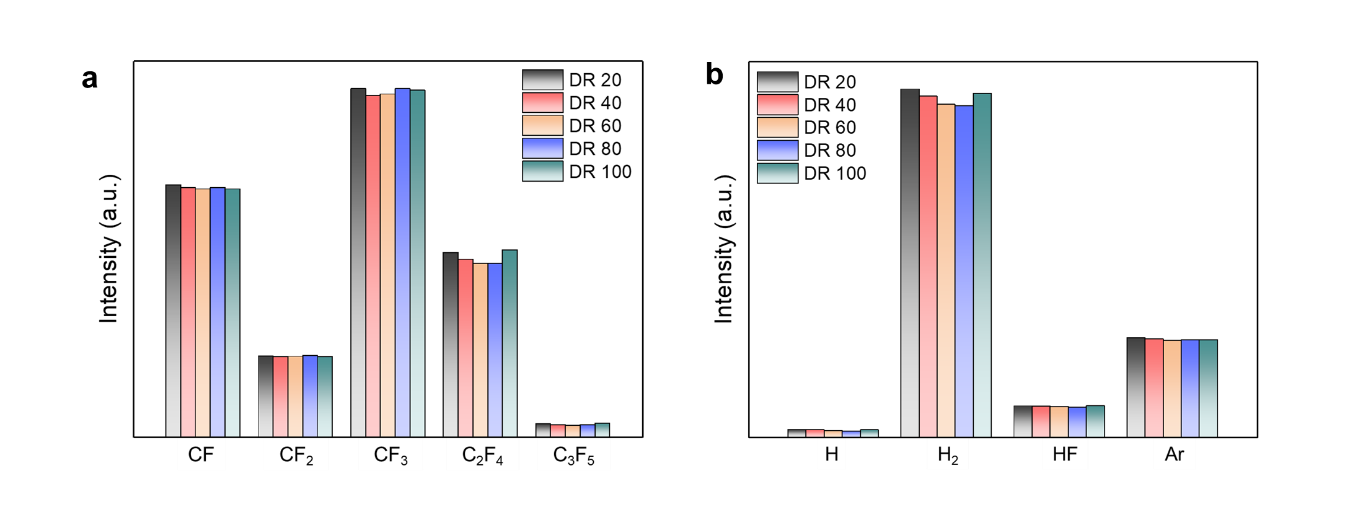


**Figure S8**. Dissociated gas composition according to pulse duty ratio in the process chamber measured using QMS for the conditions in Figure 4a. **a**, Intensities of C_x_F_y_ compositions during the etching. **b**, Hydrogen based composition and argon intensities during the etching.

When the pulse duty ratios were varied from 100 to 20 % while maintaining the Ar:H_2_ ratio at 1:3 and the CF_4_:C_4_F_8_ ratio at 1:1, no significant change in the intensity ratios among the dissociated species related to C_x_F, H_x_, and Ar in the process chamber could be observed indicating no significant variation in the ratios of dissociated gas species during the pulsing.


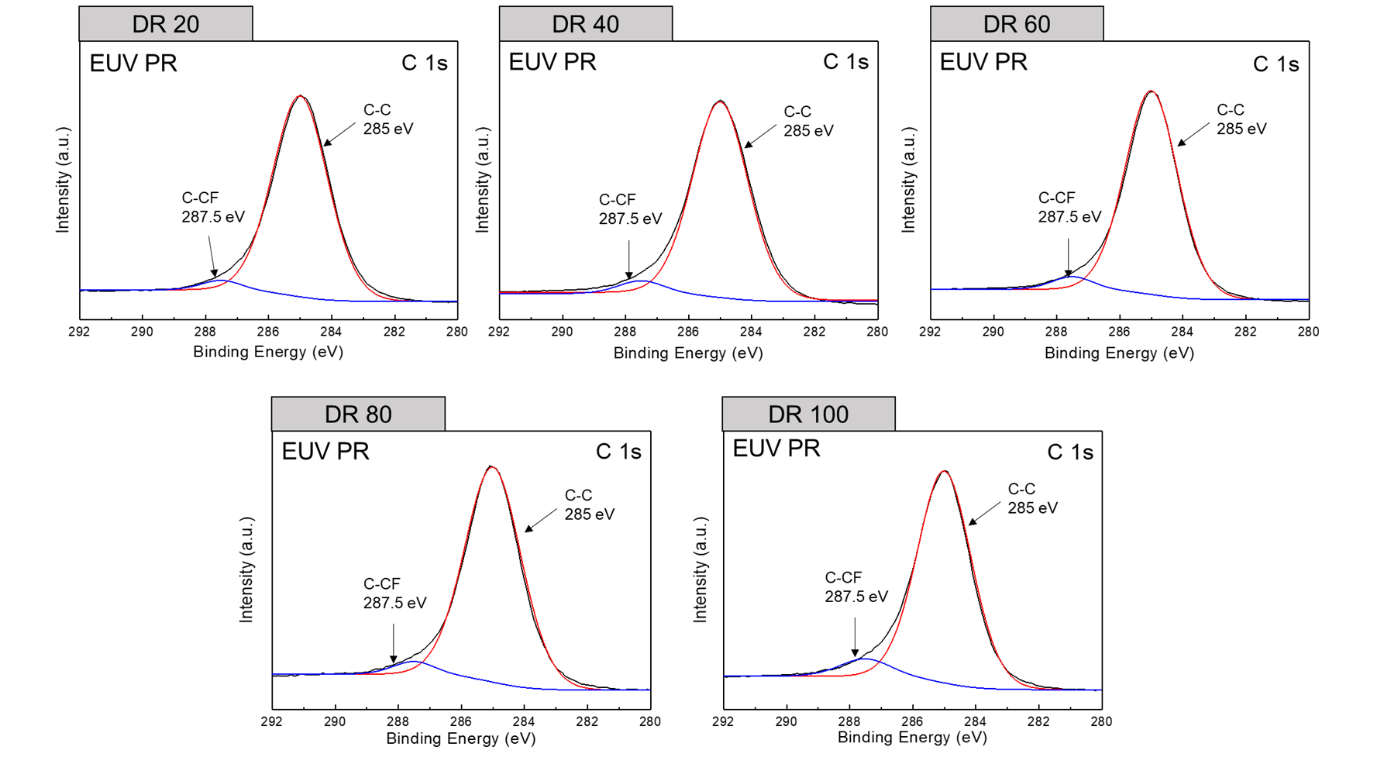


**Figure S9.** XPS C 1s binding states of EUV PR surface after etching with different duty ratio while keeping Ar:H_2_ ratio (fed to the ion beam source) at 1:3 and CF_4_:C_4_F_8_ ratio (fed to the process chamber) at 1:1.

The binding states of carbon on the EUV surface after the etching using the conditions in Fig. 5a showed the C-CF bonding at 287.5 eV in addition to C-C bonding at 285 eV, and which indicates the formation of a fluorocarbon layer on the etched EUV PR surface.


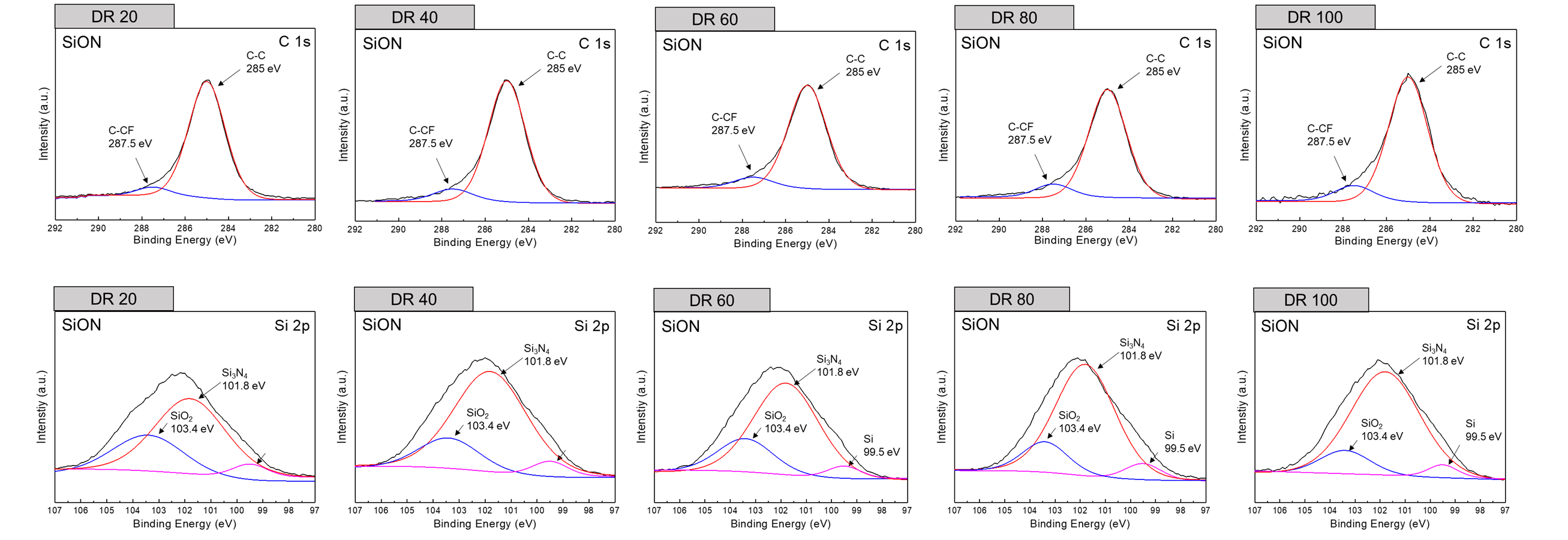


**Figure S10.** XPS C 1s and Si 2p binding states of SiON surface after etching with different grid duty ratios while keeping Ar:H_2_ ratio (fed to the ion beam source) at 1:3 and CF_4_:C_4_F_8_ ratio (fed to the process chamber) at 1:1.

The binding states of carbon and silicon on the etched SiON surface using the conditions in Fig. 5b showed the C-CF bonding in addition to C-C bonding without Si-F or Si-C bindings, which indicates the formation of a fluorocarbon polymer layer on the etched SiON surface.


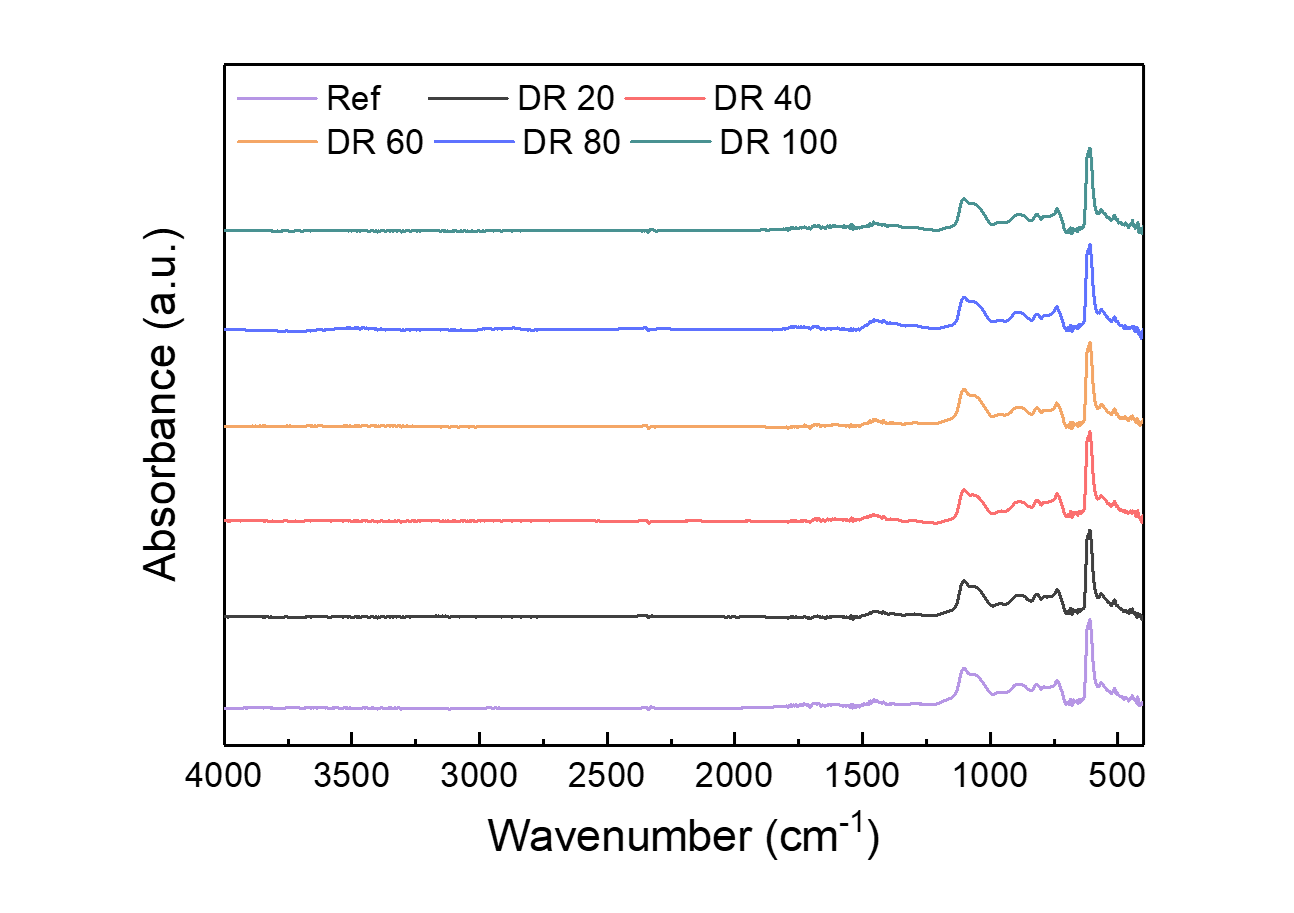


**Figure S11.** FTIR spectra of EUV PR before and after the etching using the conditions in Fig. 6a,b.

The whole FTIR spectra before and after etching using different grid pulsed duty ratios appeared to show no differences but detailed spectral investigation showed the differences in binding states. As the duty ratio is decreased from 100 to 60 %, the peak intensities related to higher carbon-to-carbon binding states such as C=C stretching vibration (1628, 1637, 2327 cm^-1^), C=C stretching vibration of benzene ring (1561 cm^-1^), and C$\equiv$C vibration (2375 cm^-1^) peak were decreased while increasing C=O stretching vibration (1728, 1745 cm^-1^) and C-H bending vibration (1462 cm^-1^) peak.


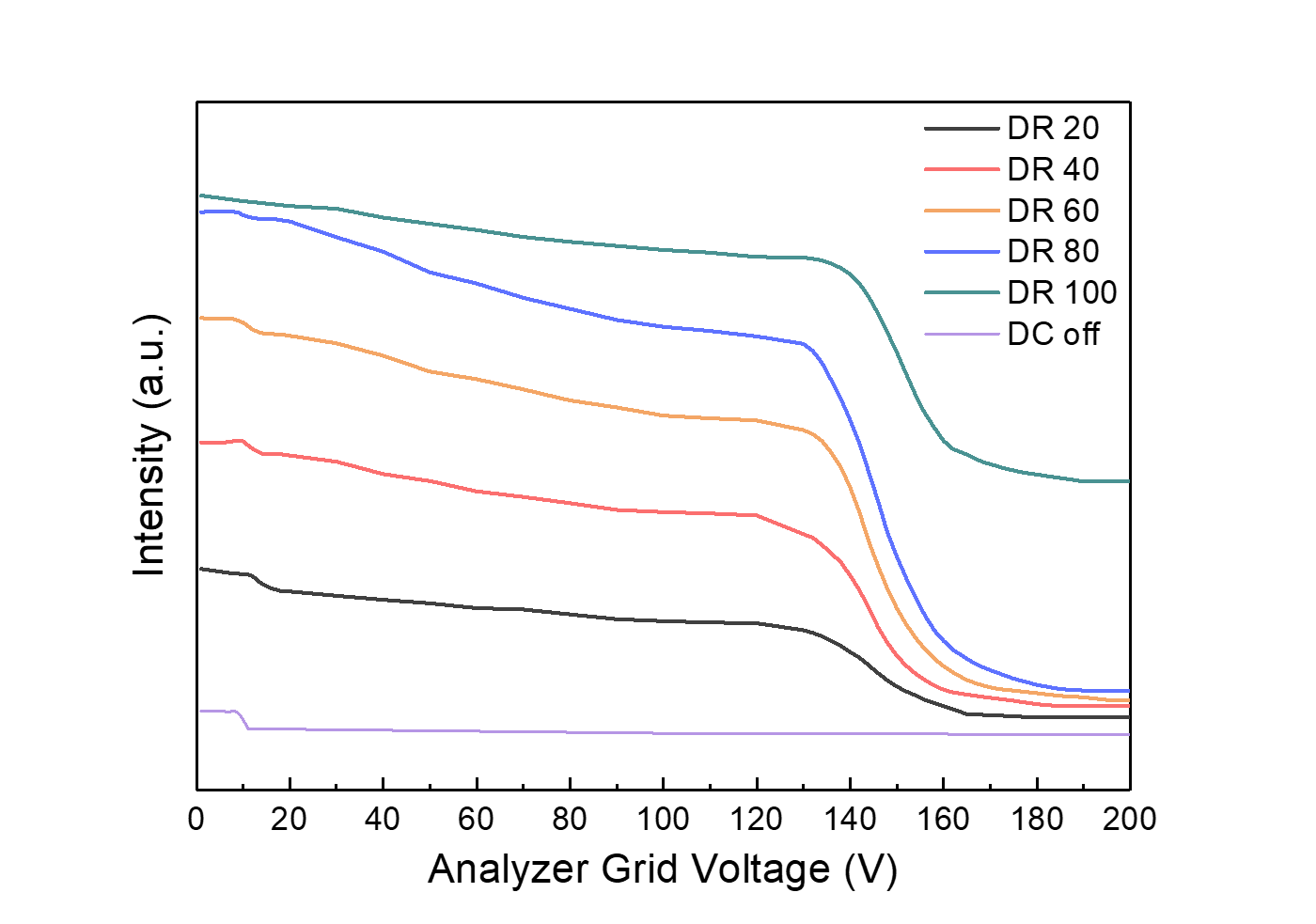


**Figure S12.** Ion beam currents to the substrate as a function of ion energy analyzer voltage for different pulse duty ratios.

For the pulse-off period, a low ion bombardment energy of ~ 10 eV related to the plasma potential from the ICP source only was observed while, for the 100 % of duty ratio, that is, for the continuous DC mode, only high energy ion beam energy ~ 160 eV (highest energy) close to the 1^st^ grid voltage (+ 150 V) + plasma potential (~10 eV) was observed. As the duty ratio is decreased from 100 to 20 %, the high ion energy peak intensity peak related to pulse duty-on period was decreased with the increase of low ion energy peak intensity related to the pulse duty-off period. The total ion current (that is, total ion bombardment flux) to the substrate was the highest for the continuous DC mode (100%) and it was decreased with decreasing pulse duty.

**Reference**

[1] Kim, S. H. & Kim, C. H. & Choi, W. J. & Lee, T. G. & Cho, S. K. & Yang, Y. S. & Lee, J. H. & Lee, S.-J. Fluorocarbon thin films fabricated using carbon nanotube/polytetrafluoroethylene composite polymer targets via mid-frequency sputtering. *Scientific reports* **2017**, *7* (1), 1451.

[2] Villamanca, D. & Colin, M. & Ching, K. & Rawal, A. & Wu, Y. & Kim, D. J. & Dubois, M. & Chen, S. Preparation and properties of graphene oxyfluoride films. *Applied Surface Science* **2024**, *646*, 158822.

[3] Yu, G. & Tay, B. & Sun, Z. & Pan, L. Properties of fluorinated amorphous diamond like carbon films by PECVD. *Applied Surface Science* **2003**, *219* (3-4), 228-237.

[4] Diéguez, L. & Caballero, D. & Calderer, J. & Moreno, M. & Martínez, E. & Samitier, J. Optical gratings coated with thin Si3N4 layer for efficient immunosensing by optical waveguide lightmode spectroscopy. *Biosensors* **2012**, *2* (2), 114-126.

[5] Ismail, E. & Sabry, D. & Mahdy, H. & Khalil, M. Synthesis and Characterization of some Ternary Metal Complexes of Curcumin with 1, 10-phenanthroline and their Anticancer Applications. *Journal of Scientific Research* **2014**, *6* (3).

[6] Kammer, S. & Albinsky, K. & Sandner, B. & Wartewig, S. Polymerization of hydroxyalkyl methacrylates characterized by combination of FT-Raman and step-scan FT-ir photoacoustic spectroscopy. *Polymer* **1999**, *40* (5), 1131-1137.

[7] Zhang, Y. & Fu, L. & Xu, W. & Zhou, J. Rapid and Efficient Microwave Catalytic Oxidation Degradation of Chlorotetracycline Over Mno2-Coo/Ac Microwave Catalyst. *Ac Microwave Catalyst*.

[8] Yu, L. & He, R. & Zhang, Y. & Gao, J. Effect of surface treatment on flexural and tribological properties of poly (p-phenylene benzobisoxazole)/polyimide composites under normal and elevated temperature. *Materials* **2018**, *11* (11), 2131.

[9] Yañez-Cruz, M. G. & Villanueva-Ibáñez, M. & Méndez-Arriaga, F. & Lucho-Constantino, C. A. & Hernández-Pérez, M. d. l. Á. & Ramírez-Vargas, M. d. R. & Flores-González, M. A. Green route synthesis and characterization of β-Bi_2_O_3_/SiO_2_ and β-Bi_2_O_3_/Bi_2_O_2_. 75/SiO_2_ using Juglans regia L. shell aqueous extract and photocatalytic properties for the degradation of RB-5. *Journal of Analytical Science and Technology* **2022**, *13* (1), 52.

[10] Takei, K.-i. & Takahashi, R. & Noguchi, T. Correlation between the hydrogen-bond structures and the C=O stretching frequencies of carboxylic acids as studied by density functional theory calculations: Theoretical basis for interpretation of infrared bands of carboxylic groups in proteins. *The Journal of Physical Chemistry B* **2008**, *112* (21), 6725-6731.

[11] Ahmad, A. & Khan, M. A. & Nazir, A. & Arshad, S. N. & Qadir, M. B. & Khaliq, Z. & Khan, Z. S. & Satti, A. N. & Mushtaq, B. & Shahzad, A. Triaxial electrospun mixed-phased TiO2 nanofiber-in-nanotube structure with enhanced photocatalytic activity. *Microporous and Mesoporous Materials* **2021**, *320*, 111104.

[12] Saikia, B. J. & Parthasarathy, G. & Borah, R. R. Investigations of organic matter in meteorites using Fourier transform infrared and micro-Raman spectroscopic methods: Implications for origin of extraterrestrial organic matter. *Jour. Indian Geophys. Union* **2022**, *26* (1), 62-77.
